# Touch, press and stroke: a soft capacitive sensor skin

**DOI:** 10.1038/s41598-023-43714-6

**Published:** 2023-10-25

**Authors:** Mirza S. Sarwar, Ryusuke Ishizaki, Kieran Morton, Claire Preston, Tan Nguyen, Xu Fan, Bertille Dupont, Leanna Hogarth, Takahide Yoshiike, Ruixin Qiu, Yiting Wu, Shahriar Mirabbasi, John D. W. Madden

**Affiliations:** 1 Electrical and Computer Engineering, Advanced Materials and Process Engineering Laboratory, University of British Columbia, Vancouver, V6T 1Z4 Canada; 2 Frontier Robotics, Innovative Research Excellence, Honda R&D Co., Ltd, 8-1 Honcho, Wako-shi, Saitama, 351-0188 Japan

**Keywords:** Electrical and electronic engineering, Biomedical engineering

## Abstract

Soft sensors that can discriminate shear and normal force could help provide machines the fine control desirable for safe and effective physical interactions with people. A capacitive sensor is made for this purpose, composed of patterned elastomer and containing both fixed and sliding pillars that allow the sensor to deform and buckle, much like skin itself. The sensor differentiates between simultaneously applied normal force and shear using summation and differences of signals from four deformable capacitors. Cross talk from shear to normal force is less than 2.5%, and between shear axes is less than 10%. Normal and shear stress sensitivity is 0.49 kPa and 0.31 kPa respectively, with a minimum displacement resolution of 40 μm. In addition, finger proximity is detectable at a range of up to 15 mm. The operation is demonstrated on a simple gripper holding a cup. The combination of features and the straightforward fabrication method make this sensor a candidate for implementation as a sensing skin for humanoid robotics applications.

## Introduction

To accommodate for complex interactions between humans and robots, it is important to design a method for touch identification that can be active on fingertips and other sensing surfaces. Ideally, the approach will be scalable to cover over most of a robot’s surface area, forming an artificial or electronic skin^1,2^. Such a technology is also sought for neurally controlled prosthetic devices to enhance motor control^3,4^. The functional requirements of an artificial skin include the ability to sense and differentiate tactile stimuli such as light touch, normal force, and shear^1^. Combined shear and normal sensing capability enables grip of fragile objects for manipulation while avoiding excessive forces^5,6^. Altun et al.^7^ emphasise the benefits of shear sensing to recognize human touch gestures on a robot, and Shehata et al.^8^ have described the benefits of feedback sensing in applications such as prosthetic limbs. Having a smooth and soft skin, rather than a hard or bumpy surface, helps make the surface more lifelike.

The objective of this work is to create a soft sensor which provides displacement signals in three axes that can be readily discriminated and may in turn be related to force. Softness or compliance in force sensors enables a more natural robot-human interaction, and three sensing axes improves dexterity over normal force alone. Soft normal and shear force sensors are typically capacitive^9–11^, piezoelectric^12^, piezoresistive^13–15^, or magnetic^16^. Flexibility is incorporated using elastomer layers and deformable electrode materials that contain silver nanowires^17^, carbon nanotubes^18–20^, liquid metals^21,22^, and hydrogels^23^. Silicone based elastomer substrates such as Ecoflex^21,24^ and PDMS^25^ have been used to make the substrate stretchable. In addition to normal pressure sensing capabilities, shear sensing is important to possess for an artificial skin. Most soft sensors do not measure both shear and pressure, and those that do often have significant drawbacks. In this work we have sought to overcome some of the limitations associated with previous normal and shear sensors including lack of discrimation of direction^26^, uneven upper surfaces needed to convert shear to torque^27^, or rigid circuitry integrated into the sensor that restrict bending, stretching, and spatial resolution^16^.

**Figure 1:**
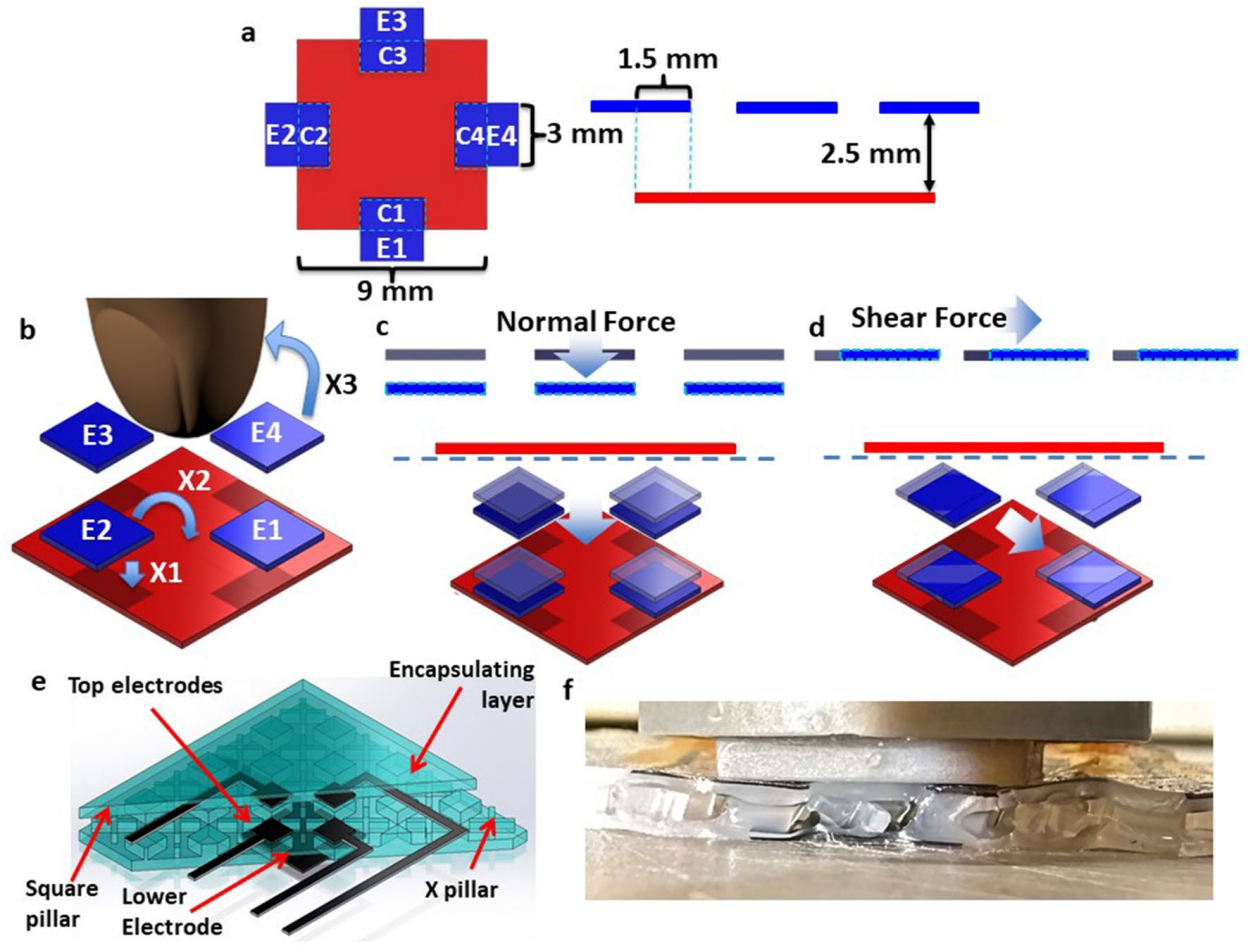
The sensor and working principle. (**A**) Top view of electrode architecture (left) and side view (right) (**B**) Sensor electrode layout showing four top electrodes (blue E1–E4) and one bottom one (red). Electric fields couple directly between the top and bottom electrodes (X1), while some fringing fields (X2, X3) extend above the plane of the device and can couple into a finger for proximity detection. The device is a mutual capacitive sensor in which in (**D**) an applied pressure displaces the top electrodes (originally grey) downwards (blue) to increase coupling with the bottom electrode (red), while (**E**) shear is detected by the lateral displacement and varying overlap of the top and bottom electrodes (dielectric omitted for clarity). In (**F**) portions of the sensor are cut away to display the structure and electrode arrangement. (**G**) Cross-section of sensor showing localized buckling upon shearing with a finger.

Several studies have been done on hard, silicon-based tactile sensors incorporating shear detection, but these tend to be too stiff or brittle to enable highly conformable sensor arrays^14,28^ and require involved fabrication processes^25^. Several soft capacitive sensors that have successfully discriminated between forces. In the present work we use an alternative electrode and dielectric geometry that increases sensitivity by an order of magnitude. The dielectric is compliant and is compressible thanks to the addition of pillars^29,30^, and the electrodes are spaced as in Fig. 1A to allow greater capacitance change. Ham et al.^11^ recently demonstrated a similar five electrode 3-axis force sensor design to our own that also detects rotation around the surface normal. Their simulation shows the benefits of using porous polymers and pillar structures as dielectrics to increase sensitivity relative to the use of a solid dielectric. They also show an estimation of combined multi-axis force inputs, with average error of about 10%. Boutry et al.^31^ demonstrated an array of internal bumps patterned with five thin capacitive electrodes. A soft, flat surface sits above these small domes, which has five perpendicular electrodes that overlap with the electrodes below. The pattern of response produced when the two layers move relative to each other enables the differentiation between simultaneous pressure and shear in this soft sensor. However, a single pressure/shear sensing location requires interpretation of data from a 5 × 5 array of capacitive sensors, which is a challenge for scalability to larger arrays, and quantitative differentiation has not yet been shown. Charalambides et al.^32^ demonstrated a solid dielectric PDMS-based capacitive shear sensor, but this device requires a significant initial normal force (1.8 N) before functioning.

Three dimensional piezoelectric, magnetic and optical sensing has also been demonstrated. A 2 × 2 array of trapezoidal PDMS protrusions on a flexible PCB were coated with piezoelectric PVDF film^33^. By comparing the voltage output from the faces of each trapezoid the direction of applied force is determined. The sensor is relatively stiff, with minimum detectable shear and normal stresses of 45 kPa and 100 kPa, respectively. These sensitivities are poor compared to the sensitivity of skin itself, where displacements of 10 µm are detected at low frequencies^34^. Xela uSkin^35^ is a commercial magnetic normal and shear force sensor that can be expanded into an array of up to 4 × 6, and is sensitive to applied forces down to approximately 10 mN or 0.45 kPa in normal force with a lateral spatial resolution of 4.7 mm per taxel. A permanent magnet is placed in a rubbery layer above a rigid Hall-effect sensing chip, which detects the deformation in the sensor surface from movement of the magnet. The underlying chip (one per taxel) adds to cost and restricts bending, limiting the curvature of the surface on which it can be mounted. Unlike skin it is not readily stretchable, and while it can bend, each taxel is stiff; these properties make it less practical for use on tightly curved surfaces such as fingertips. Choi et al.^36^ demonstrate a similar magnetic sensor that is scaled up to a large array, but this sensor requires the same stiff embedded components as the uSkin^35^ in addition to a minimum height requirement of 6 mm due to the parameters of the magnetic field adding to the thickness of the sensor. This embedded-chip system is common in sensors that are otherwise soft, which makes them difficult to use in applications requiring high bendability or stretchability.

We present a soft and stretchable sensor with the ability to sense proximity, normal force and shear. We employ capacitance addition and differences between electrodes to extract directional forces. The shear and pressure magnitudes are output as separate numeric quantities and therefore are easy to interpret and implement in automated systems. A major contribution is the demonstration of minimal coupling between axes, proving the basic architecture. The non-linear stiffness^37^ and ability to buckle (Fig. 1A left) simulates human skin—a potential advantage for creating sensor arrays that do not couple, and relevant to the desire to match constitutive behaviour of skin in advanced humanoid robots, such as that of Sophia (Hanson Robotics)^38^ or iCub (EU project RobotCub)^39^ to make more realistic human synths^40^. The sensor surface is smooth, making it more skin-like than some previous work, which uses bumps on the sensor surface to create torque^25,27,41^. The sensor also detects the approach of a finger, or in general any grounded conductor^42^ using mutual capacitance but is relatively insensitive to insulating materials. The sensor also has a simple readout circuit which provides capacitive signals through reading only four capacitances. Mutual capacitive sensing has also been found to be relatively stable to temperature change^43^.

## Results

A set of five independently connected electrodes is used for the capacitive sensing, enabling displacements and forces applied to the surface of the sensor to be resolved in 3 dimensions. Four top sense electrodes (*E1*–*E4*) are capacitively coupled to one bottom ground electrode, as shown in Fig. 1A, forming capacitances *C1*–*C4*. The top and bottom electrodes are coupled by electric fields both going directly through the dielectric (X1) and projecting outwards (X2), as shown in Fig. 1B. The capacitances between each of the top electrodes and the bottom reference are sequentially measured using an AD7745 capacitance-digital converter and a multiplexer. Connection between the soft sensor elements and this circuit is made using copper tape attached to conductive elastomer and copper leads respectively. These capacitances change in a way that depends on the nature of the stimulus.

The working principle is as follows. A human finger in proximity or lightly touching acts as a virtual ground and decouples the projected electric fields (labeled X3 in Fig. 1B), thereby reducing all the capacitances *C1*–*C4* in a similar manner^42^. Upon application of a compression force, all four top active electrodes, *E1*–*E4*, are displaced towards the bottom electrode, thereby increasing all four capacitances, *C1*–*C4* (Fig. 1C). Applying a shear force in the rightward direction moves *E3* to the right, increasing the overlapping area with the bottom electrode, and in turn increasing *C3*. *E1* also moves the to the right, decreasing overlap area with bottom electrode, in turn decreasing *C1* (Fig. 1D). The shear in this axis has minimal effects on *C2* and *C4* as their overlap with the bottom electrode does not change. This combination of changes in *C1*–*C4* is characteristic of a shear in the positive x-axis and provides information on the magnitude of the shear and its direction. The truth table of responses is presented in Table 1.

**Table 1:** Truth table for proximity/light touch, two-axis shear and normal force.

Stimulus	C1	C2	C3	C4
Light touch	Down	Down	Down	Down
Normal force	Up	Up	Up	Up
Shear + ve X direction	Down	Unchanged	Up	Unchanged
Shear − ve X direction	Up	Unchanged	Down	Unchanged
Shear + ve Y direction	Unchanged	Up	Unchanged	Down
Shear − ve Y direction	Unchanged	Down	Unchanged	Up

Mathematically we can separate the effects of shear and normal displacement from the resulting changes in capacitance. When normal displacement is applied all capacitances increase, while the shear effects are proportional to the difference in capacitances. For example, the shear displacement magnitude parallel to the *C*_*1*_, *C*_*3*_ axis is nonlinearly proportional to the expression $(C_{3} C_{1}^{\prime } - C_{1} C_{3}^{\prime } )/\left( {C_{1}^{\prime } + C_{3}^{\prime } } \right)$, where *C*_*1*_ and *C*_*3*_ are the capacitance values without force applied, and *C*_*1*_*’* and *C*_*3*_*’* are the values at the time of interest. We demonstrate that this equation is valid for shear directionality even with the application of a normal force. The shear signal in the perpendicular direction is given by a similar expression, replacing *C*_*1*_ and *C*_*3*_ with *C*_*2*_ and *C*_*4*_. Normal displacement of the dielectric is proportional to the sum of differences, $(\Delta C_{1} + \Delta C_{2} + \Delta C_{3} + \Delta C_{4} )/\left( {C_{1}^{\prime } + C_{2}^{\prime } + C_{3}^{\prime } + C_{4}^{\prime } } \right)$, where *ΔC*_*i*_ represents the change in capacitance, *C*_*i*_^*’*^*—C*_*i*_; this is shown to be valid even with a simultaneously applied shear. The overall change in capacitance of each electrode is the superposition of these individual changes, which are separated by the equations. These analytical equations are nonlinearly proportional to the strain experienced by the device, and may be mapped to three-axis applied displacement force through calibration. Derivation of the analytical equations is shown in Supplemental S1.

To obtain a larger change in capacitance, localize the effect of shear, and simulate the buckling and stretching of skin, a novel dielectric architecture is used as shown in Fig. 1E. The dielectric consists of elastomer pillars supporting the top layer and air. There are two types of alternating pillars—square, with the electrodes *E1*–*E4* located on top of them, and X-shaped. The spacing and aspect ratio of the square pillars are such that they can easily bend upon application of a shear at the top surface, making the device more sensitive compared to when a solid layer of elastomer is used. The square pillars are anchored on both the top and bottom layers, while the X pillars are only anchored to the bottom layer, as shown in Fig. 1F. This allows for a smooth top layer by preventing it from collapsing in regions not supported by square pillars while simultaneously enabling a sliding motion of the top layer upon application of a shear and gives the sensor the ability to buckle and stretch in a localized region, mimicking human skin, as seen in Fig. 1F.

**Figure 2:**
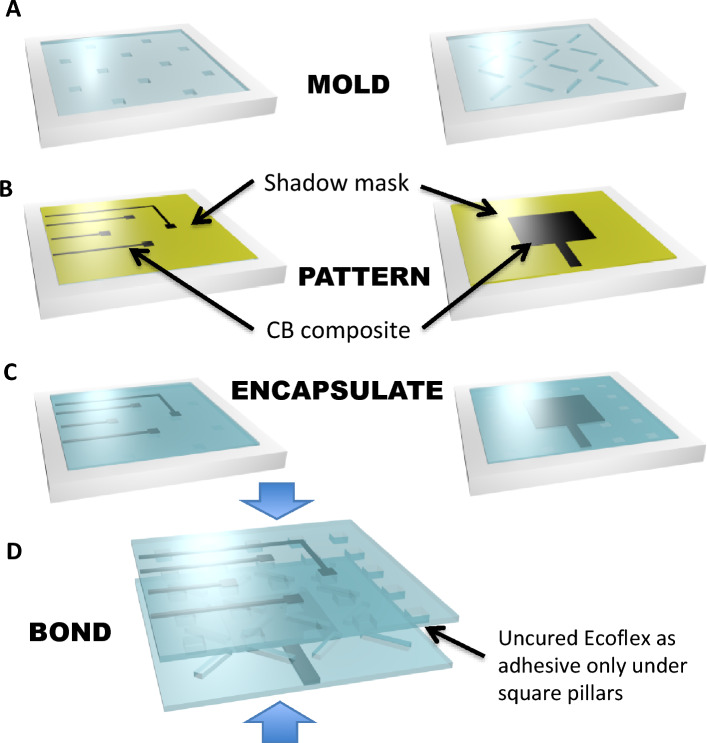
Fabrication of the sensor. (**A**) Step 1: Mold. Mold filled with Ecoflex**™**, with the square pillars that will form the top surface of the skin shown at left and the X-shaped supporsts that form the bottom layer shown at right. (**B**) Step 2: Pattern electrodes (black) using carbon black-Ecoflex™ composite by doctor blading through a shadow mask. (**C**) Spin on encapsulating layer to prevent external electrical contact. (**D**) Step 3: Bond layers using a thin layer of uncured Ecoflex™ which glues the square pillars to the base layer.

**Figure 3:**
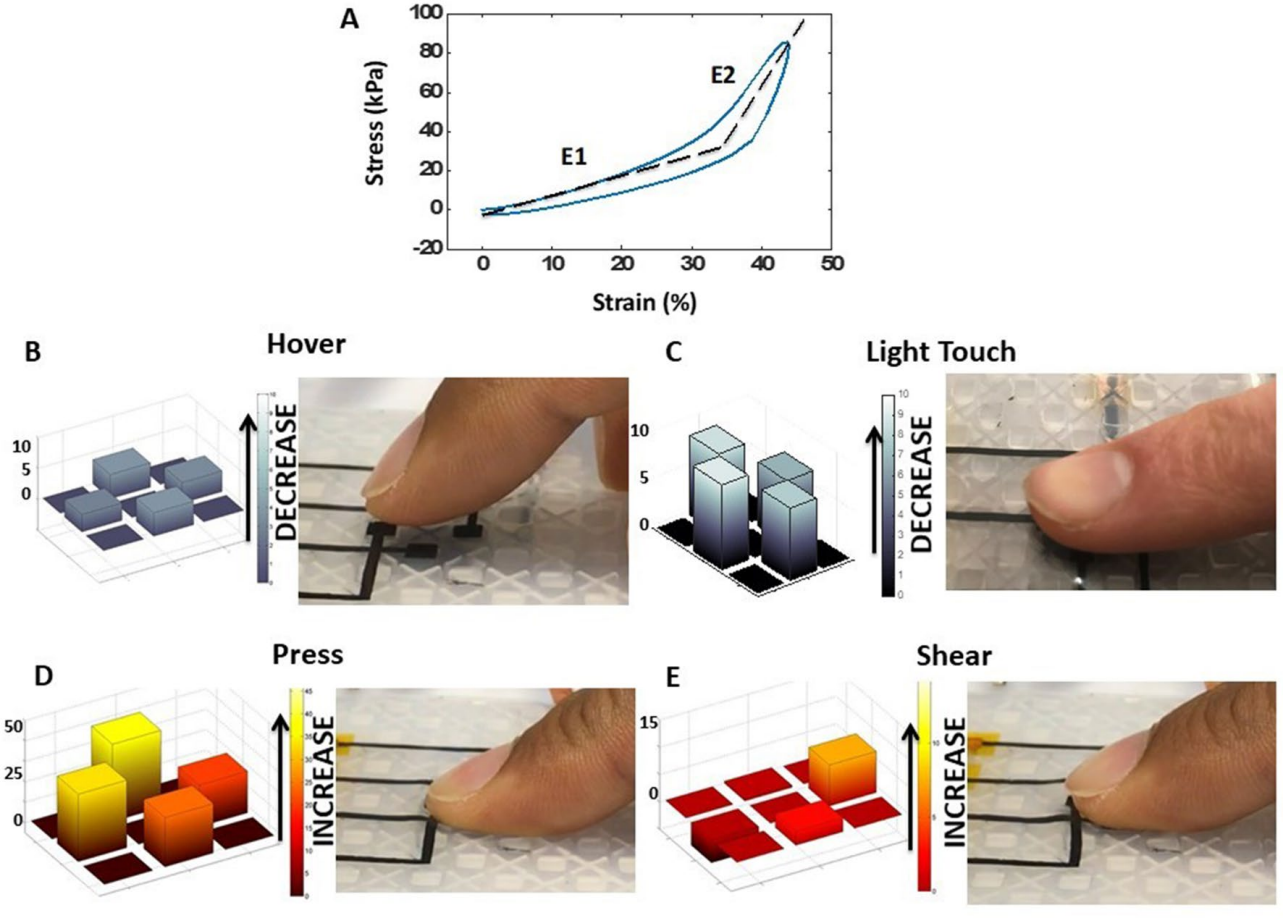
Sensor response to different stimuli. (**A**) Non-linear elastic response similar to human skin. *E1* is a smaller effective elastic modulus for small strains while *E2* is a larger elastic modulus for larger strains. (**B**) Response to a hovering finger (**C**) a light touch (**D**) a press and (**E**) a shear. All capacitive axes are in %ΔC.

The materials and methods are chosen to be low cost and appropriate for large-format mass production. A simple three-step mold-pattern-bond (MPB), Fig. 2A–D, produces a unibody sensor. The elastomer used is Ecoflex™ 00-30, which is highly compliant and has a skin-like feel. The stretchable electrodes are carbon black mixed with Ecoflex™. Although the resistivity of the carbon black based stretchable conductor is large (approximately 0.1 Ω-m), it is sufficient for low-current capacitive sensing. Ecoflex™ 00-30 is first cured in the top and bottom molds (Fig. 2A) to build the top and bottom segments, with the two types of pillars in the two molds being 1.5 mm tall, beneath a 0.3 mm thick top layer. The air pockets are 1.5 mm tall, the square pillars are 3 mm × 3 mm, and the X-pillars are 5.8 mm long on each leg. The electrodes are patterned with carbon black containing Ecoflex™ using shadow masks made from cut transparency film (Staples multipurpose transparency film, 120 μm thick) shown in Fig. 2B. The electrodes (top 4 being 3 mm × 3 mm and bottom 9 mm × 9 mm) are covered with an encapsulating layer of Ecoflex™ (Fig. 2C) approximately 300 μm thick. Half of the area of each of the top 4 electrodes overlaps with the bottom electrode midway along the four edges (Fig. 1A). A grounding layer is added to the top or bottom of the sensor using the same layering process as the sense electrodes, before the electrodes are added. The top segment is then bonded to the bottom by applying a thin layer of uncured Ecoflex™ on the bottom of the square pillars only (Fig. 2D). The sensor has an effective elastic modulus for compression of approximately 160 kPa (skin has a modulus of ~ 400 kPa^44^). A stress–strain plot for our sensor is shown in Fig. 3A, demonstrating a low effective elastic modulus *E1* at small strains and a higher modulus *E2* for larger strains, similar to human skin^37^.

Figure 3B demonstrates the sensor response to the approach and contact of a finger. The four bars in the map correspond to the four capacitances *C1*–*C4*. Upon approach, the projected field couples increasingly with the finger and the coupling between the excitation and sense electrodes will decrease along with all four capacitances *C1*–*C4* (Fig. 2B). The maps in Fig. 2B,C show the % decrease in the positive y-axis. The capacitances reach a minimum with a 10–12% decrease upon light contact, as shown in Fig. 2C. The human finger or other conducting object acts as a virtual ground, which leads to the decrease in capacitance. When approached or touched by a plastic object with a size similar to a finger, the capacitance shows a modest increase (up to 3%). The response is small and in the opposite in direction, as capacitance is increased due to the higher effective dielectric constant. The sensitivity to human approach may be valuable in robotics applications where the robot is expected to interact with humans in a delicate manner. Sensing can be done through clothing and is sensitive at a distance of 1.5 cm for electrodes of this scale, as shown below for maximum range. This ability to sense proximity could also be valuable in control, providing advance warning of a collision, or helping guide grasp.

Upon application of force normal to the sensor surface, the dielectric thickness is decreased and all four capacitances increase, as shown in Fig. 2D. Figure 2E shows the application of a shear and the sensor’s response to it. The applied shear buckles the skin on the leading edge and stretches the skin on the trailing edge. This is achieved by the dielectric architecture in Fig. 1E, which enables sliding of non-bonded surfaces, and separation when under compression—much like skin itself. The capacitance response to a shear is shown in the map in Fig. 2E. The capacitance at the trailing edge of the shear increases due to the increase in overlap area, as illustrated in Fig. 1D. Also seen in Fig. 2E is the decrease in capacitance at the leading edge of the sheared sensor as overlap with the reference is reduced. The two responses are not perfectly equal and opposite, due to the superimposed normal force that is applied during the act of shearing. The capacitances perpendicular to the axis of the shear force increase slightly due to this downward deformation.

One key ability of this sensor architecture is to simultaneously detect and estimate normal and horizontal displacements and forces. This is done using the difference and summation signals, including normalizing by base capacitance in the shear case to get a dimensionless expression, yielding:

1 $$normal \,signal = \frac{\eta }{d} = \frac{{\Delta C_{1} + \Delta C_{2} + \Delta C_{3} + \Delta C_{4} }}{{C_{1}^{\prime } + C_{2}^{\prime } + C_{3}^{\prime } + C_{4}^{\prime } }},$$

2 $$shear \,signal \,along\, C_{1} \left\langle - \right\rangle C_{3} = \frac{\lambda }{d} = \frac{1}{\varepsilon W}\frac{{C_{3} C_{1}^{\prime } - C_{1} C_{3}^{\prime } }}{{C_{3}^{\prime } + C_{1}^{\prime } }},$$

and

3 $$shear \,signal \, along\, C_{2} \left\langle - \right\rangle C_{4} = \frac{\lambda }{d} = \frac{1}{\varepsilon W}\frac{{C_{4} C_{2}^{\prime } - C_{2} C_{4}^{\prime } }}{{C_{4}^{\prime } + C_{2}^{\prime } }}.$$

**Figure 4:**
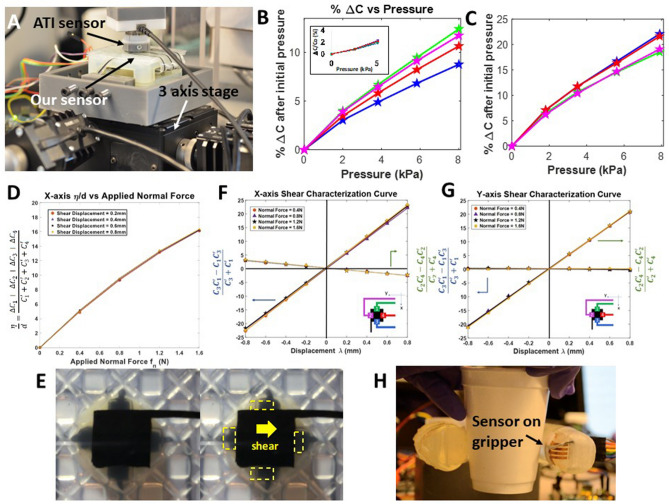
Characterization of sensor. (**A**) Characterization setup showing the ATI Nano 17 load cell and the Thorlabs 3-axis stage. (**B**) Plot showing relation between applied pressure and %ΔC change in capacitance. The inset shows the simulated %ΔC change in capacitance with pressure. (**C**) Plot showing relation between applied pressure and % change in capacitance with GND shielding to reduce parasitic capacitance. (**D**) Plots showing response of sensor to normal forces with simultaneous horizontal forces being applied. (**E**) Image from the bottom of the sensor at steady state (left) and with a shear force applied (right). (**F**) Plots showing response of sensor to horizontal shear displacement in the X-axis with simultaneous normal forces being applied. (**G**) Plots showing response of sensor to horizontal shear displacement in the Y-axis with simultaneous normal forces being applied. (**H**) Sensor applied on a robotic gripper holding a paper cup.

These equations assume uniform displacement of all four electrodes, *E1*–*E4*, relative to the base electrode. The derivations of the equations are shown in the supplementary S1. This model assumes that the electrodes deform in a uniform manner due to applied force, and that the indentation object has flat dimensions of at least the size of the shear sensing area (1.1 × 1.1 cm). Simulations are conducted in COMSOL, shown in Fig. S2 to verify the normal or lateral motion for normal or shear forces respectively. Based on these simulated results, the model should provide a nonlinearly proportional calculation of applied forced directionality. This set of three equations separates the shear induced changes in capacitance along the two horizontal axes, and the normal strain induced strain capacitance. In order to estimate applied force from capacitance, the calibration curves shown in Fig. 4 are used. Using the resultant force vectors in the x- and y-directions, force vectors can be estimated.

Like commercial force sensors, including the ATI Nano 17 multi-axis force sensor used here for calibration, the new soft sensor is able to differentiate between simultaneously applied shear and normal components. To demonstrate this, measured shear and normal forces obtained from the ATI Nano 17 are compared to those predicted by our capacitive sensor. A 14 × 14 mm square indenter printed from resin (*Formlabs*) is used to indent the sensor surface, and displacements are applied using a Nanomax 3-axis stage. Indenter size and shape is chosen to cover the entire sensor surface and provide a uniform response, shown in Fig. 4A. Normal force and % change in capacitance (Δ*C*/*C*_*0*_) for the four capacitive electrodes are plotted in Fig. 4B. We see change in capacitance increase with increasing applied force for all four electrodes, as is expected given that the separation between capacitor plates is decreasing. The slopes of the responses flatten with increasing force, consistent with the increasing stiffness of the sensor (Fig. 2A). There is significant variation between electrodes in the Δ*C*/*C*_*0*_ magnitude and slope, shown in Fig. 4B. When looking instead at raw change in capacitance, the changes are more consistent, shown in Fig. S3. Parasitic coupling due to trace asymmetry leads to a difference in baseline capacitance (*C*_1_ = 44 pF. *C*_2_ = 49 pF, *C*_3_ = 49 pF, *C*_4_ = 38 pF). Using the ground shield architecture shown in Fig. S7reduces the variation between electrodes, seen in Fig. 4C. The ground shielding lowers the baseline capacitances and also brings the electrode capacitance closer to each other (*C*_1_ = 14 pF, *C*_2_ = 13 pF, *C*_3_ = 14 pF, *C*_4_ = 15 pF). The sensitivity is 2.8%/kPa for low pressures. A light press using a fingertip is approximately 50 g, which corresponds to a normal force of about 0.5 N and is detectable. The force ranges characterized in both normal and shear are chosen to match the lower forces observed in humanoid touch^5,6^ to focus on the low forces associated with dexterous manipulation. The sensitivity drops with increasing load, Fig. S13,S14, consistent with the increasing stiffness of the structure, to 0.3%/kPa at 80 kPa. The sensor also demonstrates a consistent response to repeated small-force normal characterization, with a maximum change in force response of 0.593% and maximum change in signal response of 2.93% across a five-peak characterization set. The sensor also exhibited minimal wear from characterization performed and practical tests, including movie S1–S3 with practical indentation objects.

To illustrate the sensor’s ability to detect normal force independent of shear forces applied, the normal signal calculated using Eq. (1) is plotted against the applied normal strain. The summation of the responses of the four electrode capacitances results in a single curve relating applied normal strain and capacitance, as shown in Fig. 4D. This curve remains approximately constant under simultaneous horizontal (shear) forces of varying magnitude. All four curves associated with different levels of shear force coincide with each other within 2.5%. The normal force detection is independent of the level of shear force applied, showing that reliable normal force readings can be obtained, independent of shear. When normal strains are in excess of 30%, significant viscoelastic responses are observed, as seen in Fig. S5. However, the measurements are stable relative to applied shear strain, as shown in Figure S6. This suggests, as expected, that capacitance is determined by electrode spacing, not by forces between electrodes. Minimum normal forces detected are 0.095 N (0.49 kPa) with a SNR of 5 dB, corresponding to a normal strain of 1.6%. Assuming accuracy decreases as sensor deformation becomes further from the linear region, and worst-case accuracy occurs at the lowest measured sensitivity, the worst-case normal force accuracy of the sensor is ± 0.04 N.

In order to characterize the shear force sensitivity, a horizontal force is applied by moving the Nanomax 3-axis stage in either the X or Y axes. Upon application of shear, the surface of the sensor buckles, similar to human skin, as shown in Fig. 1F. Images looking up through the bottom of the sensor in rest and sheared states, are shown in Fig. 4E. The calculated shear displacement from the sensor is plotted against the applied shear displacement in Fig. 4F,G for displacements along the X-axis and Y-axis. There are four sets of plots in each figure corresponding to different normal forces applied simultaneously to the shear force; agreement between curves in this plot indicates the independence of sensing directions from one another. The X and Y axis shear are nearly independent, with some X–Y cross-over seen in Fig. 4F (10% maximum). This is likely due to imperfect shielding, leaving some coupling between electrode E2 and E4 with the adjacent traces. Minimum shear forces detected are 0.061 N (0.31 kPa) with a SNR of 5 dB, corresponding to shear strain of 1.8%, showing a sensitivity of ~ 0.3% change in capacitance per change in strain (or ~ 20% change in capacitance per Newton, and 5% change/kPa), with a maximum force tested of 0.8 N (4.1 kPa), corresponding to a shear strain of up to 53%. Based on the same assumptions of worst-case accuracy as the normal force response, the shear force accuracy of the sensor is ± 0.043 N.

Using results from the X-axis shear magnitude and Y-axis shear magnitude, the resultant shear magnitude and angle can be estimated, as demonstrated in Movie S1. A strawberry was used to apply forces to the sensor. The strawberry, despite being relatively soft, was not physically degraded by the interactions with the sensor, which is important for applications that require a gentle interaction^45^. The sensitivity to shear forces is also illustrated in Fig. 4H and Movie S2. Here the sensor is implemented on a gripper, which is used to grab a Styrofoam cup. The sensor is mounted on a flex printed circuit board. As shown in Fig. S8, the flex PCB contains the four sense electrodes (*E1*–*E4*) and all the readout hardware. The hardware is concealed inside the gripper, as shown in Movie S2. The video demonstrates the sensor’s ability to detect the shear forces as the cup gets heavier due to the addition of water. A key offering of the sensor is the ability to detect shear changes as the cup becomes heavier, which requires multi-axis sensitivity. This feature is a step towards mass estimation and slip detection, and in turn dextrous manipulation of objects. Movie S1, shows the sensor response to the application of forces in 3 axes, showing the output signal as a shear vector and normal magnitude.

**Figure 5:**
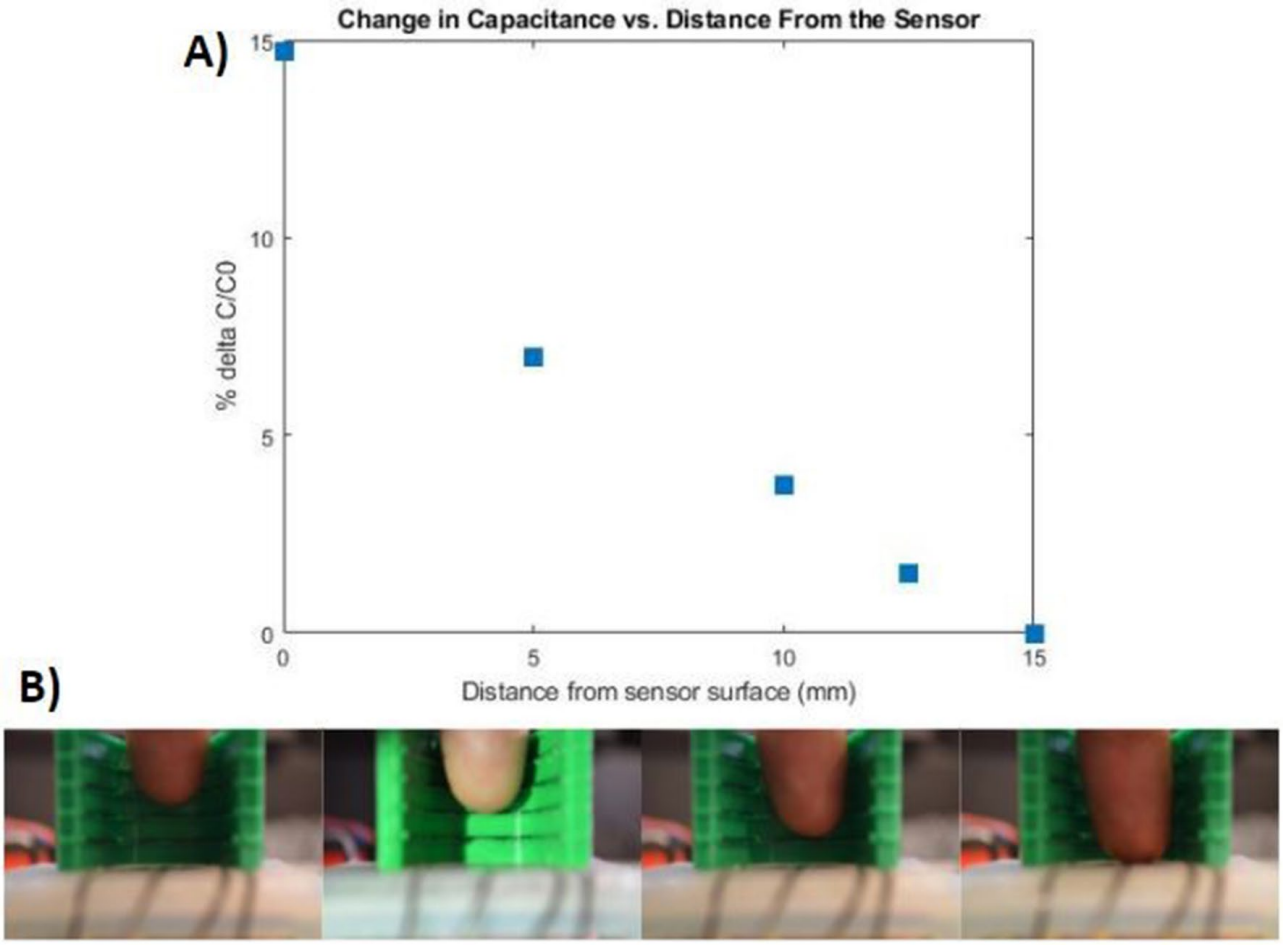
Approaching object proximity characterization. (**A**) Plot showing average taxel capacitive response to an approaching finger with 15 mm distance as baseline capacitance (**B**) Finger approaching sensor within a plastic distance-measuring guide.

Proximity to the sensor of a grounded object produces a clearly detectable signal out to a distance of 15 mm. This range is observed through the approach of a finger towards the surface of the sensor, acting as an approaching cylindrical grounded object. The response of the sensor to the approach of a finger from a maximum distance of 15 mm is illustrated in Fig. 5A, with the approaching finger shown in Fig. 5B. Due to the electric field siphoning by the approaching finger, a decrease in capacitance occurred up to a maximum average ∆C/C_0_ of 14.7%, measured across the four upper taxels of the sensor and using the capacitance reading at 15 mm as the baseline. This proximity could be used to enable anticipation of incoming objects, and identification of light touch on the sensor surface with very low normal force.

Although shear, proximity and touch can all be detected, there is a region of ambiguity between pressure and proximity. An approaching conductor causes the capacitance to decrease, and vice versa on retraction. The same response is seen if the finger approaches and pushes very lightly on the sensor, with capacitance rising after contact due to compression of the capacitors, shown in Fig. S9. To resolve this ambiguity between light pressure and proximity a grounded conductor plane is added on top of the sensor, removing the mutual capacitive coupling between the electrodes and the approaching object or person. In order to retain the ability to detect proximity the conductor plane is multiplexed to a self-capacitance readout IC (FDC1004), illustrated in Fig. S10. Movie S3 demonstrates the sensor detecting approach, light touch, and normal force, combined shear and normal force. There is no effect of proximity on force detection, and vice-versa. The configuration allows detection through clothing, and the registration of contact that is below the resolution of the normal and shear force sensors.

## Discussion

The soft capacitive sensor distinguishes normal force from shear with direction discrimination based on additive or differential capacitive signals. It also enables proximity detection, and has skin-like properties. The mechanoreceptors of human skin remain more sensitive, detecting forces as small as ~ 30 mN^46^, three quarters the weight of a sheet of paper, and corresponding to a pressure of ~ 300 Pa—so there is still work to do to match this sensitivity. The dielectric architecture allows plenty of room for scaling up or down, however. Changes in sensitivity can be achieved by using materials of different intrinsic stiffnesses, as has been explored by Ham et al.^11^, as well as by varying the geometry and size of the features—creating the promise of both smaller and/or softer, higher resolution sensors, and bigger and/or stiffer, higher force versions. An attractive feature of any capacitive technology is that its spatial resolution scales in proportion to the dimensions of the electrodes. On the larger scale, previous work^42^ suggests that the active area can be hundreds of square meters, which can be low in cost due to the inexpensive materials employed.

Next steps in sensor development include coupling the sensors with control to enable the manipulation of delicate objects. There is also the potential to fabricate and test arrays of sensors to form a full skin, and enable large-area skin sensing. Effective scaling to arrays would require a reconfiguration of the sensor design described here to maximize density of sensing taxels while keeping the number of connections required manageable. One potential approach to this scaling is to arrange the taxels into grids of overlapping taxels, where vertical and horizontal traces could be shared across taxels, as demonstrated in normal force sensing^47^. Success in this area would enable robots and prosthetics to interact more effectively with their environments.

## Materials and methods

### Sensor fabrition

A FormLabs Form2 Printer was used to print two molds (Grey V4 material). Ecoflex™ 00-30 (Smooth-On two-part silicone elastomer) was mixed in a 1 (part A):1 (part B) mass ratio for 3 min and degassed to remove air bubbles. The mixture was poured into the molds and levelled with a glass slide, degassed once more for 2 min and placed in an oven at 60 °C for 10 min.

Masks for patterning the electrodes were laser cut from Staples multipurpose transparency sheets (120 μm in thickness). Each mask was manually aligned with the dielectric patterns on top of the cured Ecoflex mold layers while still in the mold, with the 4-electrode pattern laid down on the square-pillar piece and the bottom electrode laid down on the X-pillar dielectric. Carbon black (H30253 Carbon Black Super P® Conductive, Alfa Aesar) was mixed in a Thinky ARE-310 mixer with Ecoflex 00-30 in a 2(carbon black):10(part A):10(part B) mass ratio for 4 min, then spread over the aligned mask exposed regions to cover the electrode region. A glass slide was used to level the conductive paste and remove excess material before liftoff of the mask, leaving the patterned electrode.

Ecoflex 00-30 uncured mixture was prepared using the same mixing and degassing method as above and spin-coated at 300 RPM on top of the patterned electrodes in the molds for 60 s to achieve an encapsulating layer of ~ 400 μm thickness. The molds were again placed in the oven for 10 min to cure the top layer.

The two molded pieces with patterned and encapsulating electrodes were peeled off of the molds. A 1:1 Ecoflex mixture was prepared and spin-coated onto a petri dish at 300 RPM for 60 s. The square-pillar layer was laid gently pillar-side-down on top of this uncured layer and lifted off to form an adhesive layer on the bottom of the pillars. The dielectric piece with X-pillars was placed X side up and the square pillar piece was manually aligned such that the pillars fell in the center of the gaps between the crosses and pressed down to adhere the two layers and form the full sensor. The sensor was cured in the oven for 10 min at 60 °C to finish the curing process.

### Sensor readout

An Analog Devices CDC chip (AD7745) was used to read the capacitance. The CDC has a similar working principle to a Delta-Sigma analog-to-digital converter (ADC). One multiplexer (MAX 4518) was used to cycle through the four top electrodes, and the output of the multiplexer was fed to the CDC. The microcontroller (Arduino Mega 2560) controlled the multiplexer and CDC and fed the final data through a serial port into a computer where digital values of the capacitance could be acquired and displayed. For the self-capacitance augmentation an FDC1004 CDC is used in addition to the AD7745.

### Sensor characterization

The sensor is placed on a box containing the readout circuit, and is fixed on a 3-axis stage (Thorlabs NanoMax), as is shown in Fig. 4A. A 14 mm × 14 mm 3D printed cover is attached on to the ATI load cell, acting as the indenter plate that applied force to the sensor. Four levelling screws are used to ensure the ATI sensor is flat relative to the surface of the sensor, as any tilt will bring about unwanted torque. The soft sensor is lifted up to contact the ATI sensor using the stage. Characterization is initiated when the ATI sensor is just touching the test sensor and applying a small force (typically 0.1 N).

### Supplementary Information

Supplementary Information 1.

Supplementary Video 1.

Supplementary Video 2.

Supplementary Video 3.

## Data Availability

All data needed to evaluate the conclusions in the paper are present in the paper and/or the Supplementary Materials. Additional data related to this paper may be requested from the authors, specifically M.S.
