# Supplementary material for: Touch, press and stroke: a soft capacitive sensor skin
Source: Sci Rep. 2023 Oct 25;13:17390. doi: 10.1038/s41598-023-43714-6 (PMC10600100; doi:10.1038/s41598-023-43714-6)
Supplement: Supplementary file 1 — Supplementary Information 1. [file 41598_2023_43714_MOESM1_ESM.docx]

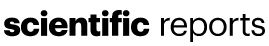


Supplementary Materials for

Soft artificial skin for detecting approach, contact and shear

Mirza S. Sarwar^1^*, Ryusuke Ishizaki^2^, Kieran Morton^1^ Claire Preston^1^, Tan Nguyen^1^, Xu Fan^1^, Bertille Dupont^1^, Leanna Hogarth^1^, Takahide Yoshiike^2^, Ruixin Qiu^1^, Yiting Wu^1^, Shahriar Mirabbasi^1^, John D.W. Madden^1^*

*Corresponding author. Email: mirzas@ece.ubc.ca, jmadden@ece.ubc.ca

This PDF file includes:

Supplementary Text

Figs. S1 to S11

Other Supplementary Materials for this manuscript include the following:

Movies S1 to S3

Supplementary Text

1 Analytical Equations to Differentiate Between Shear and Normal Displacements

Capacitance signals are measured are between each of the four sense electrodes, *E1* to *E4*, depicted in blue in Figure 1A of the main text, relative to the bottom electrode, shown in red, forming capacitances *C1* to *C4*. Given the signal from any one electrode, it is impossible to distinguish shear from compression, as both have an effect. However, we have four capacitance signals, so it should be possible to extract more information. If there is a combined shear and normal force applied, we should be able to determine the three unknown displacements from these four values. In order to separate shear displacement and normal displacement from the sensor output, we propose analytical equations for shear displacement of each axis that are intended to be independent of the normal displacement and, similarly, and an equation for the normal displacement that is intended to be independent of the shear displacement. As discussed in the main text in the results section, shear displacement will lead to displacement tangential to the surface, and create a differential signal, while compression will create a common change in capacitance amongst all four capacitances. One approach is to measure shear and normal displacements, and create calibration curves. Here we first scale the common and difference equations, guided by a parallel plate model. The assumption that the parallel plate model holds under applied force is supported by simulations, discussed in S1.3. This approach and the resulting measurements show that we can separate shear from normal force, and largely measure shear independently in each axis.

Fig. S1 is a cross-sectional drawing of the position of the sensor electrodes under a combined applied normal and shear force, and is used to guide the development of equations 1, 2 and 3 in the main text (S10 and S5 below). The derivation conducted here assumes displacement by an indenter with a flat area greater than 11 mm x 11 mm, creating equal tangential and normal displacements in all four top electrodes. The shear and normal signals are predicted to be independent of each other based on Figures 4 D, F and G. Normal force signal is independent of shear force, and shear axes are largely independent. Calibration factors or look up tables can then be used to relate position to force. or simulations used to find resultant forces from the applied displacements according to the sensor structure.

1.1 Separating shear displacement from normal displacement

The objective in producing equations (1), (2) and (3) presented in the main text is to establish differences and summations of capacitive signals that separate shear in each axis from normal displacements. A parallel plate model is assumed to help establish scaling that will make the axes independent. The parallel plate model underestimates the full capacitive coupling between electrodes since, given that the separation between plates (2.5 mm) is significant compared to the overlap distance (1.5 mm). Nonetheless the simple model includes the key drivers of change in capacitance – the separations between electrodes and their overlap. In this model, the undeformed state (Fig. S1 left) is characterized by a distance, *d*, between the top plates (red) and the bottom plate (blue), and an overlap area, *A* (dashed boxes in Figure 1A). Starting with a one-dimensional picture, the left and right capacitances (*C_1_* and *C_2_*) are then equal, and estimated to be:

$C_{1}=\frac{\varepsilon A}{d},C_{2}=\frac{\varepsilon A}{d}$. (S1)

Here, the permittivity is *ε*. When a shear displacement, *λ*, and a simultaneous compressive normal displacement, *η*, are applied, there is both a change in overlap area and distance between plates. Neglecting fringing fields, and changes in permittivity due to bulging or bending of the pillars, the new capacitances are:

$C_{1}^{'}=\frac{\varepsilon\left( W\times\left( L+\lambda\right) \right)}{d-\eta},C_{2}^{'}=\frac{\varepsilon\left( W\times\left( L-\lambda\right) \right)}{d-\eta}$ . (S2)

*W* is the width of the electrode, into the page. The separation between plates following deformation is computed for the left and right capacitances:

$\left( d-\eta\right)=\frac{\varepsilon\left( W\times\left( L+\lambda\right) \right)}{C_{1}^{'}}$ (S3), and

$\left( d-\eta\right)=\frac{\varepsilon\left( W\times\left( L-\lambda\right) \right)}{C_{2}^{'}}$ . (S4)

Assuming uniform displacement across the taxel (S3) and (S4) are set to be equal, resulting in the equation:

$\frac{\epsilon WL}{C_{1}^{'}}+\frac{\epsilon W\lambda}{C_{1}^{'}}-\frac{\epsilon WL}{C_{2}^{'}}+\frac{\epsilon W\lambda}{C_{2}^{'}}=0$ ,

which is then multiplied by 1/*d* to obtain:

$\frac{C_{1}}{C_{1}^{'}}+\frac{\epsilon W\lambda}{dC_{1}^{'}}-\frac{C_{2}}{C_{2}^{'}}+\frac{\epsilon W\lambda}{{dC}_{2}^{'}}=0$ _._

This is rearranged to give:

$\frac{\epsilon W\lambda}{d}\left( \frac{1}{C_{1}^{'}}+\frac{1}{C_{2}^{'}} \right)=\frac{C_{2}}{C_{2}^{'}}-\frac{C_{1}}{C_{1}^{'}}$ _._

The resulting estimated shear strain is then:

$shear strain\to\frac{\lambda}{d}=\frac{C_{2}C_{1}^{'}-C_{1}C_{2}^{'}}{\epsilon W\left( C_{2}^{'}+C_{1}^{'} \right)}$ . (S5)

This is Equation (2) in the main text. Equation (3) for the second axis is derived in the same way. The shear depends on the difference between capacitances, as expected.

1.2 Separating normal from shear displacement

In order to derive Equation (1), the sum of all four capacitances is used. Using a parallel plate capacitance once again, the four original capacitances are:

$C_{1}=\frac{\epsilon WL}{d},C2=\frac{\epsilon WL}{d},C_{3}=\frac{\epsilon WL}{d}, \mathrm{and} C_{4}=\frac{\epsilon WL}{d}$. (Eqn. S6)

The four capacitances in the deformed state are:

$C_{1}^{'}=\frac{\epsilon W\left( L+\lambda\right)}{d-\eta},C_{2}^{'}=\frac{\epsilon W\left( L-\lambda\right)}{d-\eta},C_{3}^{'}=\frac{\epsilon WL}{d-\eta},{\mathrm{and} C}_{4}^{'}=\frac{\epsilon WL}{d-\eta}$. (Eqn. S7)

The sum of the initial and deformed capacitances are given by:

$C_{1}+C_{2}+C_{3}+C_{4}=\frac{4\epsilon WL}{d}$ and,

$C_{1}^{'}+C_{2}^{'}+C_{3}^{'}+C_{4}^{'}=\frac{4\epsilon WL}{d-\eta}$. (Eqn. S8)

Taking the ratio of these two sums, and rearranging,

$\frac{d-\eta}{d}=\frac{C_{1}+C_{2}+C_{3}+C_{4}}{C_{1}^{'}+C_{2}^{'}+C_{3}^{'}+C_{4}^{'}}$, or

$\frac{\eta}{d}=1-\frac{C_{1}+C_{2}+C_{3}+C_{4}}{C_{1}^{'}+C_{2}^{'}+C_{3}^{'}+C_{4}^{'}}$. (Eqn. S9)

The strain in the dielectric is then:

$\frac{\eta}{d}=\frac{C_{1}^{'}-C_{1}+C_{2}^{'}-C_{2}+C_{3}^{'}-C_{3}+C_{4}^{'}-C_{4}}{C_{1}^{'}+C_{2}^{'}+C_{3}^{'}+C_{4}^{'}}$, or

$normal strain\to\frac{\eta}{d}=\frac{\Delta C_{1}+ \Delta C_{2}+\Delta C_{3}+\Delta C_{4}}{C_{1}^{'}+C_{2}^{'}+C_{3}^{'}+C_{4}^{'}}$. (Eqn. S10)

S10 is Equation 1 in the main text.

Not included in the equations are the effects of changing effective dielectric constant as the sensors are displaced, the effect of differences in dimensions between capacitors, and the effects of fringing. There is also an assumption that displacements are uniform across the taxel. The relatively simple model is nevertheless effective in largely separating shear from normal force.

1.3 Simulated Electrode Response to Normal and Shear Displacements

Simulations are conducted in COMSOL in both the normal and shear directions. These are used to help estimate distortions of the electrode configuration and pillars that result from deformation. Results are shown in Fig. S2. The mechanical simulations are designed to resemble the characterization conducted on the sensor, with a 14 mm x 14 mm square indenter imparting normal and shear forces on the sensor surface. This model uses a Mooney-Rivlin 2-parameter hyperelastic material model with C_10_ = 7212.7914 Pa and C_01_ = 2590.1961 Pa based on fitting to bulk material stress-strain curves. Normal displacement up to 0.6 mm (18% strain) and shear displacement up to 1.5 mm (45% strain), shown in Fig. S2B and C, are achieved on the sensor, and the resulting 3D displacement on the sensor electrodes are observed. These strains are up to 187.5% of the strains involved in characterization conducted. These simulation results show the applicability of the parallel plate model to the forces characterized. Non-deformation effects on the electronic structure of the sensor capacitors such as changing dielectric constant or small changes in electrode dimensions are also relegated to these empirical equations.

Under simulated normal strain the upper and lower electrodes remain approximately parallel, shown in Fig. S2B, with some deformation at larger strains due to the compression of the underlying pillars. The displaced upper electrodes are separated from the stiff indenter by a 0.3 mm layer of elastomer only, causing the indenter to effectively prevent lateral deformation of the electrodes. As the indenter applies normal strain, the dielectric pillars are compressed and experience lateral expansion due to the Poisson effect. This lateral expansion also affects the sensor electrodes, which are expected to experience a small amount of Poisson expansion. At a normal strain of 18% there is some deformation of the sensor electrodes but little distortion of electrode shape. The electrodes experience 4.4% lateral expansion due to compression, which affects the parallel plate capacitance for the analytical equations but does not invalidate a parallel plate model basis. Some tilt was also observed from the outside to inside edges of the electrodes, up to 1.3%. Overall, there is a small amount of deformation and electrode tilt imparted by normal strains in the simulations conducted. Therefore, the parallel plate approximation is expected to hold reasonably well at the normal displacements observed in characterization.

Simulations in the shear direction are conducted by applying up to a 1.5 mm shear displacement, shown in Fig. S2C; the square pillars and upper have been significantly deformed due to shear, and the upper electrodes have been shifted relative to the lower layer of the sensor. In the shear direction, it is seen that the upper electrodes remain relatively stable in terms of 3D deformation from their original shape. The sheared electrodes experience 4.8E^‑3^ mm (0.16% of electrode width) of stretch at 1.5 mm displacement, demonstrating minimal distortion of the electrode shape. There is tilting in the electrodes observed under shear which does not affect the shape of the electrodes themselves but would influence the parallel-plate nature of the architecture. This tilting is caused by the pillars in the dielectric layer, which experience a small amount of tilt due to the applied shear resulting in vertical displacement in the upper layer. Tilt observed across the sheared electrodes is 5.49E^‑2^ mm (18.3% of electrode height) at a maximum shear strain of 45%. These simulations suggest that for the large shear and normal displacements examined there is significant tilt but relatively little distortion of the electrodes. This means there may be some distortion of the parallel-plate model the analytical equations are based on at large shear strain, which should be accounted for in calibration.

2 Change in Capacitance with Normal Force

2.1 Absolute change in capacitance

Fig. S3 shows the magnitude of absolute change of the capacitance with pressure. It is observed that all four capacitors coincide relatively well. In Fig. 4B on the other hand, the four ∆C/C_0_ responses are quite scattered. This is because, although the change in capacitance is similar for all four, the baseline values are different, and so the % change values are also different. This scattering is dramatically reduced by adding ground shielding, reducing the variation between base capacitances, as seen in Fig. 4C.

2.2 Sensitivity

In order to obtain the sensitivity over a region, a straight line is fit, as shown in Fig. S4. Based on the slope, the sensitivity at this level of stress is 1.5 %/kPa. The sensitivity is highest at small strains, and drops with increasing load.

2.3 Mechanical characterization

A series of stress-strain tests of varying amplitudes were applied to the sensor to observe its mechanical properties. A displacement-controlled sine wave of 0.1 Hz is applied. The stress vs. strain curves for different values of displacement amplitude are plotted in Fig. S5.

The apparent hysteresis between the loading and unloading parts of the cycle at large strain amplitudes suggests a viscoelastic behavior of the structure. The increase in stiffness at large amplitudes shows the non-linear response of the structure. The effective elastic modulus is higher for larger strains. A similar non-linear behavior in elastic modulus with strain is observed in human skin, shown by Delalleau *et al.* [21].

Plotting ∆C/C_0_ against applied strain for a range of different displacements is shown in Fig. S6. There is very little apparent hysteresis even at large strains, showing that the capacitance is largely determined by displacement. This is expected, since capacitance is a function of geometry.

3 Ground shield to reduce parasitic coupling with interconnecting trace

There is capacitive coupling between electrical connections and the sense electrodes, as depicted in Fig. S7A. The coupling also occurs between interconnects, as discussed in part 3 above. A ground shield architecture is shown in Fig. S7B that reduces this coupling. The resulting relative change in capacitance then becomes more consistent between electrodes, as shown by comparing Fig. 4B (unshielded) and Fig. 4C (shielded).

4 Flex-Stretch sensor implementation

In order to enable higher electrode resolution and relative alignment, a flex printed circuit board can act as a base for the sensor, as shown in Fig. S8. The electrodes are now a part of the readout hardware, also providing a shielded path for the connections when a multilayer board is used.

5 Ambiguity due to proximity and small force

Combining both projected (mutual capacitance) and overlap capacitance in the same sensor leads to a small region of ambiguity, as explained in Fig. S9. In Case A, when a finger comes in close to a sensor (proximity) there is a small decrease in capacitance. The magnitude of the capacitance reaches its minimum value when a light contact is made. Retraction of the finger leads it to rise again. If the finger is still nearby, the capacitance will still be lower than the baseline value. This lowering can be interpreted as finger proximity. In Case B, when the finger presses down slightly, there is a small increase in capacitance due to the decrease in the dielectric thickness. The capacitance change looks identical to a proximity response. There are cases when proximity and a light press cannot be distinguished.

In order to resolve this ambiguity, a ground plane can be used to shield the effects of the presence of a finger near the sensor. Although this solves the ambiguity issue, the sensor is now only able to detect normal and shear force. The proximity sensing ability is removed.

In order to reintroduce the proximity sensing ability, we now multiplex the top layer between a ground connection (when reading the normal/shear force response) and a self-capacitance readout IC (e.g. FDC1004), which can detect the proximity/light contact of a finger. A diagram of this architecture is shown in Fig. S10, with the upper ground plane preventing outside fields from causing sensor ambiguity.

6 Sensor response of individual electrode capacitance to shear

Fig. S11 shows the response of individual electrode capacitances of the sensor when a horizontal displacement or force is applied with a simultaneous normal force. It is observed that all four plots of %ΔC/C_0_ (under various normal forces) coincide with each other. This means that the shear response is largely independent of normal force applied. The x-axis shear response is also decoupled from shear in the y direction. In case of %ΔC/C_0_ with shear force, we see a small deviation in the case of the smallest normal force applied (i.e. 0.4N).


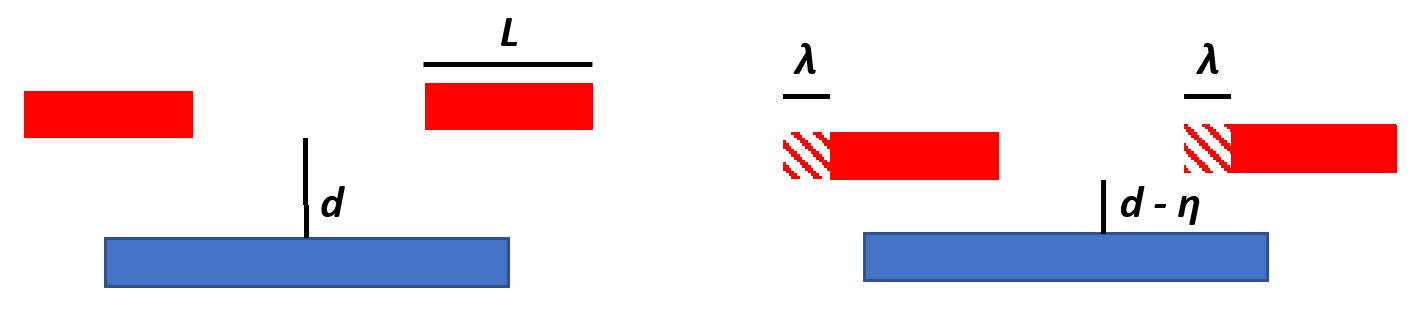


Fig. S1. Calculating sensor response with normal and shear displacement applied.


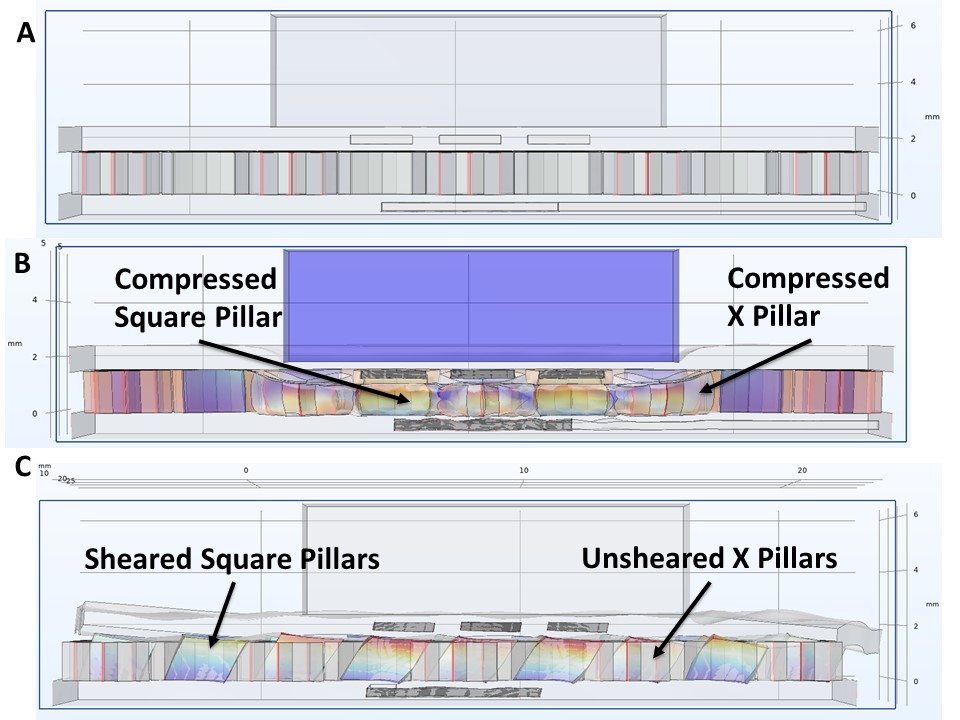


Fig S2. COMSOL structural simulation cross-section of deformations proportionally described by the theoretical model presented with electrodes colored in black, upper and lower Ecoflex layers colored white, A) model at rest with 14 x 14 mm square indenter, B) sensor model with 0.3 mm normal direction indentation (9.1% strain) applied, C) 1.5 mm shear displacement (45% strain) applied. Square and X-shaped pillars are colored using different gradients to improve visibility.

Fig. S3. Absolute change in capacitance VS pressure applied.

Fig. S4. ∆C/C_0_ *vs*. pressure showing sensitivity of about 1.5%/kPa in this case.


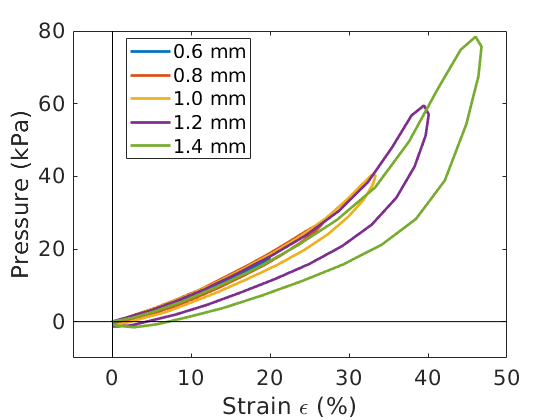


Fig. S5. Pressure vs. strain for a range of displacements


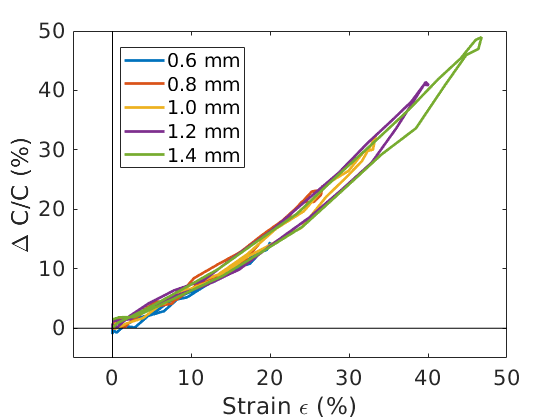


Fig. S6. Measured change in capacitance with strain.

Fig. S7. (A) Cross-section of sensor showing parasitic coupling of interconnected traces (left and right bottom) with top electrode. There is also coupling between top and bottom connection traces. (B) A ground shield is introduced to block parasitic coupling with interconnected trace, reducing the overall capacitance of the sensor, and increase the relative change in capacitance.

Fig. S8. (A) Flex-PCB with bottom electrodes of the sensor as copper pads on the PCB, and (B) Top part of the sensor with stretchable dielectric and top carbon composite electrodes bonded on bottom electrodes on flex-PCB (C) 3D model of sensor on a robot fingertip.

Fig. S9. Ambiguity of capacitive response between proximity and light contact. Case A (top): proximity leads to a decrease in capacitance, which becomes smallest upon light contact. The capacitance rises again upon partial retraction. Case B (bottom): contact followed by the application of force, which brings the capacitor plates closer, leads to increase in capacitance. The two steps in red look the same to the sensor, creating an ambiguity.

Fig. S10. Conductor plane multiplexed to FDC1004 for self-capacitive proximity sensing. The ambiguity presented in Fig. S9 can be resolved by shielding the sensor from proximity, and separately detecting proximity using the top electrode.

Fig. S11. Sensor response to shear displacement and force. The plots show that sensitivity to shear displacement (left) and shear force (right) are essentially unaffected by normal force. The x-axis shear is decoupled from the y-axis. The forces in the legend represent the constant normal forces applied during shear tests. The horizontal axes are shear displacement (left) and shear force (right). The vertical axis is relative change in capacitance.

Movie S1. Measurement of normal and shear force using the sensor, with force applied by a soft strawberry.

Movie S2. Change in normal and shear force applied by a cup to a robotic finger as a result of filling the cup with water.

Movie S3. Sensor differentiation of proximity, touch, normal force, and shear force.
